# Beyond blood glucose targets: integrating glucose variability into acute glycemic care in French ICUs - a multicenter prospective study

**DOI:** 10.1016/j.aicoj.2026.100193

**Published:** 2026-10-28

**Authors:** Suzanne Goursaud, Toufik Kamel, Amandine Thierry, Laetitia Bodet-Contentin, Nahila Himer, Vanessa Zinzoni, Paul-Henry Wicky, Julien Lorber, Guylaine Labro, Anne-Astride Bourion, Sarah Noir, Evelina Ochin, Nathalie Regnier, Thibault Noel, Célina Morand, Adrien Auvet, Aude Garin, David Schnell, Vanessa Jean-Michel, David Joganah, Constance Grignon, Emmanuelle Mercier, Bertrand Hermann, Claire Dupuis, Laurent Poiroux, for the SRLF Trial Group

**Affiliations:** a Department of Medical Intensive Care, Caen University Hospital, Caen, France; b Normandie Univ, UNICAEN, INSERM UMRS U1237 PhIND, Caen, France; c Medical Intensive Care, Orléans University Hospital, Orléans, France; d Médecine Intensive Réanimation, Centre Hospitalier Universitaire de Tours, Paris, France; e Réanimation Polyvalente, Hôpital Nord Franche-Comté, Trévenans, France; f Intensive Care Department, La Roche-sur-Yon Hospital, La Roche-Sur-Yon, France; g Medical and Infectious Diseases Intensive Care Unit, AP-HP, Bichat Hospital, Paris Diderot University, Paris, France; h Department of Intensive Care Medicine, Hospital of Saint-Nazaire, Saint-Nazaire, France; i Service de Médecine Intensive-Réanimation Groupement Hospitalier Régional Mulhouse Et Sud Alsace, Hôpital Emile Muller, Mulhouse, France; j Réanimation, Centre Hospitalier de Cholet, Cholet, France; k Service de Médecine Intensive Réanimation, Centre Hospitalier Universitaire de Besançon, Besançon, France; l Service de Réanimation Polyvalente et d'Unité de Surveillance Continue, Hôpital Simone Veil, Eaubonne, France; m Intensive Care Unit, Ouest Parisien Private Hospital, Ramsay Générale de Santé, Trappes, France; n Association Hospitalière Sainte Marie, Centre Hospitalier Sainte Marie de Clermont-Ferrand, Clermont-Ferrand, France; o Service de Médecine Intensive-Réanimation, CHU Poitiers, France; p Intensive Care Unit, Dax Hospital, Dax, France; q Service de Réanimation Polyvalente, Centre Hospitalier de Dreux, Dreux, France; r Médecine Intensive Réanimation, Centre Hospitalier Régional Universitaire (CHRU) de Tours, France; s Service de Réanimation Médicale et Maladies Infectieuses, Hôpital Chatiliez, Tourcoing, France; t Service de Reanimation, Centre Hospitalier de Haguenau, Haguenau, France; u Department of Anesthesiology, CHU Nantes, Université de Nantes, Pôle Anesthésie-Réanimation, Service d'Anesthésie Réanimation Chirurgicale, Hôtel Dieu, Nantes, France; v Service de Médecine Intensive Réanimation, CHU de Tours, Tours, France; w Inserm 1266, Institute of Psychiatry and Neurosciences of Paris, Université Paris Cité, Paris F-75014, France; x Medical ICU, Hôpital Européen Georges Pompidou, Assistance Publique-Hôpitaux de Paris (AP-HP), Centre-Université Paris Cité, Paris, France; y Department of Medical Intensive Care, Clermont-Ferrand University Hospital, Clermont-Ferrand, France; Université Clermont Auvergne, Human Nutrition Unit, INRAe, CRNH Auvergne, Clermont-Ferrand, France; z University of Angers-University of Rennes, Inserm, EHESP, Irset (Institut de Recherche en Santé, Environnement et Travail), UMR-S 1085, SFR ICAT, Angers, France

**Keywords:** Glucose variability, Hypoglycemia, Hyperglycemia, Glycemic control, Insulin, Intensive care

## Abstract

**Background:**

Although hyperglycemia is a common adaptative response in hospitalized intensive care units (ICUs) patients, there are no recommendations regarding blood glucose management protocols. Consequently, practices differ between ICUs. We aimed to prospectively describe glucose management in French-speaking ICUs with the main objective to assess the impact of glucose management on glucose variability within the first 48 h of insulin infusion.

**Results:**

A total of 650 patients from 38 ICUs across France and Belgium were included with 649 patients analyzed. Management with tighter glucose targets did not result in an increase in Glycemic Lability Index (OR 0.79, 95% CI [0.51–1.21]; *p* = 0.28) but episodes of severe hypoglycemia were more frequent in tight blood glucose control group. Nurse workload did not impact glucose variability significantly. Deviations from the protocol were frequent in 24.9% of cases, independently to the blood glucose target used. Percentage spent in the target range was significantly higher in the liberal group (*p* < 0.01). ICU mortality rate was higher in patients who had at least one episode of hypoglycemia <3.9 mmol/L (*p* = 0.04), and lower in patients with at least one episode of hyperglycemia >11.1 mmol/L (*p* = 0.04).

**Conclusions:**

Despite heterogenous practices through French speaking ICUs, management with tight blood glucose targets did not significantly impact glucose variability but was associated with higher incidence of severe hypoglycemia.

## Background

Insulin resistance and hyperglycemia is common in intensive care units (ICU) patients, whether diabetic or not [1]. Hyperglycemia reflects the acute response to stress during critical illness [2,3], and has long been associated with poor clinical outcome [[4], [5], [6]]. While uncontrolled glycemia despite prolonged insulin requirement is associated with mortality, the impact of insulin resistance and hyperglycemia alone on mortality remains uncertain [7].

Insulin therapy is widely used in ICUs to control blood glucose levels [8]. Nevertheless, there are important inter-individual variabilities not only in glycemic levels in response to an acute stress, but also in response to insulin therapy [9]. Despite numerous clinical studies on glucose control in ICU, the optimal glycemic target in critically ill patients remains unclear and debated [10]. A first large-scale study carried out in 2001 among ICU patients suggested morbidity and mortality benefits of an intensive insulin therapy aiming at tight glycemic control (maintenance of blood glucose at a level between 4.4 and 6.1 mmol/L (0.8 and 1.1 g/L)) [11,12]. However, these results have never been confirmed by subsequent large randomized trials, questioning the benefit of such strict protocols compared to more liberal ones [[13], [14], [15], [16]].

Although insulin therapy protocols are recommended in ICU, their implementation and glycemic targets remain heterogeneous and insufficiently standardized [17,18]. Recent literature has highlighted an increased risk of hypoglycemia with protocols aiming at overly restrictive glycemic targets [[17], [18], [19], [20]]. Consequently, experts suggest against titrating an insulin infusion to a lower blood glucose target 4.4–7.7 mmol/L (0.8–1.39 g/L) as compared with a higher blood glucose target range 7.8–11.1 mmol/L (1.4–2 g/L) to reduce the risk of hypoglycemia. Beyond hypoglycemia, high glucose variability (GV) has emerged as an independent risk factor for adverse outcomes in critically ill patients [21,22]. Interestingly, both hypoglycemia and GV have been well documented as being independently associated with increased mortality [23]. Management with a tight blood glucose target increases the occurrence of hypoglycemic episodes and could therefore increase GV. However, and to our knowledge, no study has investigated this link between glycemic targets and GV even though reducing GV might represent an important goal in glucose management for ICU patients.

As the literature remains controversial, clinical practices differ between ICUs depending on the centers and caregivers. Similarly, modalities of glucose monitoring and insulin administration vary across units. Moreover, since glucose management requires nursing time, its intensity may impact nursing workload, particularly in the context of inappropriate patient-to-nurse (P/N) ratio, which is itself associated with the occurrence of adverse events [[24], [25], [26]] and nosocomial infections [[27], [28], [29], [30]]. These associations may be partly attributed to an increase in missed care when P/N ratio becomes too high [19,[31], [32], [33]]. Such complications are more pronounced in ICU than in conventional hospital wards due to the higher patient volume and the urgent nature of care. However, to our knowledge, the relationship between P/N ratio and GV has not been studied in ICU [34,35].

In this context, we conducted a multicenter prospective observational study to examine glucose management practices in French and Belgian ICUs. The main objective was to assess the impact of glucose management on GV. Additionally, we described current glucose management strategies implemented in the participating ICUs and their association with nurse workload.

## Materials and methods

### Study design

The GINGER study was a multicenter observational prospective cohort study conducted in ICUs across France and Belgium (ClinicalTrials.gov: NCT05775770, registered on April 2023).

### Ethics

The study received approval in January 2023 from the *Comité de Protection des Personnes Sud Est V*. Each participant, or their legal representative, was informed about the study, and a record of non-opposition from patients or their representatives was collected.

### Study population

Inclusion criteria were (1) hyperglycemia requiring insulin therapy within 48 h of ICU admission, (2) beginning of insulin therapy within 12 h of inclusion, and (3) expected ICU stay of at least 48 h. Patients were excluded if they (1) were moribund, (2) were admitted for ketoacidosis, hyperosmolar coma or symptomatic hypoglycemia, (3) required insulin to treat hyperkaliemia, (4) presented severe acute hepatocellular failure, (5) were <18 years old, (6) were pregnant or breastfeeding women or (7) were under legal protection measures.

### Data collection and study design

Demographic data, comorbidities, cause of admission and severity score at the time of enrollment were collected. Within the first 48 h of inclusion, data were collected prospectively whenever a care procedure related to blood glucose control needed to be performed (glucose measurement, insulin dose, glucose administration). During this 48-h period, care workload, protocol deviation, glucose intake and nutrition were concomitantly collected at each glucose control procedure. Additionally, recourse to organ support (mechanical ventilation, renal replacement therapy and vasopressors), mortality, ICU and hospital length of stay were collected for each patient. Glycemic management protocol modalities had to be completed only once per center.

### Outcomes

The primary objective was to assess the association between glucose management protocols and glycemic variability, defined primarily by the Glycemic Lability Index (GLI) and secondarily the coefficient of variability (GluCV).

Secondary objectives were to assess the associations between glucose management protocols and five additional outcomes: (1) the occurrence of hypoglycemia (safety criteria), (2) nursing workload related to the protocol, (3) adherence to and deviation from the protocol, (4) protocol efficacy in achieving predefined glycemic targets, and (5) morbidity (ICU length of stay, organ supports) and mortality in ICU.

### Definitions

In our study, GV was defined based on two indices [36]:

- GLI, which quantifies the variation in differences between consecutive glucose levels, was defined as:

$$GLI=\;\frac{{\text{Σ}(\text{Δ}glycemia)}^{2}}{\frac{hours}{week}}$$

with Δ the differences between consecutive glycemia measurements (in mmol/L).

- GluCV was defined by the ratio of the standard deviation to the mean:

$$GluCV=\;\frac{\frac{\sqrt{\sum_{i=1}^{n}({{glycemia}_{i\;}-\bar{glycemia})}^{2}}}{n-1}}{\bar{glycemia}}\;x\;100$$

where $\bar{glycemia}$ is the mean of the n glucose measurements.

Adverse events were defined by the occurrence of moderate and severe hypoglycemia. Moderate hypoglycemia was defined by a glucose level < 3.9 mmol/L (0.7 g/L) and severe hypoglycemia by a glucose level < 2.2 mmol/L (0.4 g/L).

Hyperglycemia was defined by a glucose level >11 mmol/L (2 g/L), and severe hyperglycemia by a glucose level >16.5 mmol/L (3 g/L).

Three glucose target profiles were a priori distinguished based on thresholds from previous literature [19,31,33]:

- Tight control: blood glucose upper range <7.7 mmol/L (<1.4 g/L)
- Standard control: blood glucose upper range ≥7.8 mmol/L and <10 mmol/L (1.4 and 1.8 g/L)
- Liberal control: blood glucose with upper range ≥ 10 mmol/L (≥1.8 g/L).

Nursing workload was subjectively assessed at each glucose management procedure by the bedside nurse using a visual analog scale ranging from 0 (no workload) to 10 (maximum perceived workload).

Deviation from the protocol was reported by the nurse at each intervention by a qualitative variable (yes/no).

Protocol efficacy was assessed by the time required to reach the glycemic target after initiation of insulin therapy, and the time spent within prespecified glucose target ranges.

### Statistical analysis

We performed a descriptive analysis of the data, presenting continuous data as medians with interquartile ranges and categorical data as frequencies and percentages. Comparisons were carried out using Wilcoxon rank-sum tests for continuous variables, and chi2 tests or Fisher's exact tests for categorical variables.

Correlation between variables, i.e: indices of glycemic levels, GV and potential explaining factors, were explored using Pearson's correlation coefficient and represented by a correlation matrix.

We used an ordinal logistic regression model with a cumulative logit link (proportional odds model) to study the factors associated with increased GLI value. Analyses were performed with SAS software version 9.4 (SAS Institute Inc., Cary, North Carolina) and R software version 4.3.0 (R Foundation for Statistical Computing, Vienna, Austria).

## Results

### Patients’ characteristics

From March 2023 to March 2024, a cohort of 650 patients from 38 ICUs across France and Belgium met inclusion criteria. One patient withdrew consent a posteriori, leading to a total of 649 patients analyzed (Table 1). Respiratory conditions were the most common cause of admission (43%), followed by cardiovascular (14.9%) and neurological (12.5%) conditions. Comorbidities were dominated by diabetes—predominantly type 2—affecting 47.1% of patients, followed by cardiovascular diseases (25.0%) and chronic respiratory failure (24.3%).

**Table 1:** Baseline characteristics.

Characteristics	Median [IQR]/N (%)
Number of patients	649
Age	68 [60; 74]
Sex (Male)	427 (65.8)
BMI (kg/m²) (missing = 2)	27.4 [23.9; 32]
Location before ICU admission	
Continuous monitoring unit or other ICU	37 (5.7)
Emergency department / or direct admission from home	438 (67.5)
Hospital ward	165 (25.4)
Medical and rehabilitation care	9 (1.4)
Type of admission	
Medical	547 (84.3)
Urgent surgery	63 (9.7)
Scheduled surgery	39 (6)
Main reason for admission	
Respiratory	379 (43)
Neurological	81 (12.5)
Cardiovascular	97 (14.9)
Digestive / hepatic	57 (8.8)
Urological / nephrological	37 (5.7)
Metabolic / endocrinological	9 (1.4)
Haematological	12 (1.8)
Gynaecological-obstetric	3 (0.5)
ENT	9 (1.4)
Other	65 (10)
Main comorbidities	
Cardiovascular	162 (25)
Chronic respiratory failure	158 (24.3)
Immunosuppression	107 (16.5)
Chronic liver failure	48 (7.4)
Chronic renal failure	117 (18)
With dialysis	20/117 (17.1)
Diabetes	306 (47.1)
Type 1 diabetes	32/306 (10.5)
Type 2 diabetes	274/306 (89.5)
HbA1C (missing = 236)	7 [6; 8]
Diet	143/306 (48.6)
Oral treatment of diabetes	215/306 (70.3)
Insulin therapy	135/306 (44.1)
Severity on admission	
SAPS II	47 [34; 60]
SOFA	6 [4; 9]
SOFA respiratory >2	232 (35.7)
SOFA neurological >2	204 (31.4)
SOFA cardio-vascular >2	286 (44.1)
SOFA liver >2	7 (1.2)
SOFA kidney >2	100 (15.4)
SOFA coagulation >2	26 (4)

BMI: body mass index, ICU: intensive care unit, ENT: Ear, Nose, Throat, SAPS II: Simplified acute physiology score; SOFA: Sequential Organ Failure Assessment.

### Overview of glucose management in French-speaking ICUs

Among the 38 ICUs involved in this study, 36 questionnaires were collected concerning glucose management in each center (Table S1). All but one of the 36 ICUs (97.5%) had a formal protocol regarding glucose management. Glycemic targets were specified in 31 (86%) protocols and could be categorized as tight (4, 13%), standard (9, 29 %) and liberal (18, 58.1 %). Frequencies of glucose measurements varied between ICUs protocols.

### Glycemic monitoring and management during study protocol

Median glycemia at inclusion was 10.5 [8.3; 13.9] mmol/L (1.9 g/L). The percentage of time spent within the prescribed glycemic target was 39.4% [20.3; 65.3] (Table 2**)**. Glucose dysregulation was frequent, with 83.2% and 26.3% of patients experiencing at least one episode of hyperglycemia >11.1 mmol/L (2 g/L) and severe hyperglycemia >16.7 mmol/l (3 g/L) respectively. Hypoglycemia was less frequent, with 12.5% of patients with at least one episode of hypoglycemia <3.9 mmol/L (0.7 g/L) and 2.8% with severe hypoglycemia <2.2 mmol/l (0.4 g/L). Within the 48 h following inclusion, a total of 10 355 glucose measurements were collected in the 649 patients, with a median of 14 measures [12; 19] per patients. Eighty-seven percent were performed at the capillary site and 10.1% at the arterial site (Table S2). During the 48 h study protocol, 69.5% of patients received enteral nutrition including 11.1% in whom nutrition was temporarily interrupted (Table S3). Corticosteroid administration was reported in 280 (43.3%) patients. The tight control, standard control and liberal control target profiles gathered 82 (12.6%), 118 (18.2%) and 449 (69.2%) patients respectively (Table 3). Density of distribution of blood glucose values according to these 3 targets are illustrated in Fig. 1.

**Table 2:** Characteristics of blood glucose management at the patient level.

Characteristics	Median [IQR]/N(%)
Glycemia at inclusion (mmol/L)	10.5 [8.3; 13.9]
Median glycemia per patient (mmol/L)	9.4 [8.3; 10.5]
Min glycemia during ICU stay (mmol/L)	6.1 [5; 7.2]
Max glycemia during ICU stay (mmol/L)	13.9 [11.7; 17.2]
Safety	
At least one hypoglycemia (<3.9 mmol/L)	81 (12.5)
At least one severe hypoglycemia (<2.2 mmol/L)	18 (2.8)
At least one hyperglycemia (> 11.1 mmol/L)	540 (83.2)
At least one severe hyperglycemia (> 16.7 mmol/L)	171 (26.3)
Glucose variability indices	
GLI ([mmol/L]^2^/hour/week)	1.5 [0.7; 2.9]
gluCV	24.6 [17.9; 33.2]
% time in the range (prescribed glycemic target)	39.4 [20.3; 65.3]
Insulin administration modalities	
At least once insulin IV	454 (70)
At least once insulin bolus (subcutaneous or intravenous)	335 (51.6)
At least once insulin long acting	42 (6.5)
Dose insulin /24 h	30 [13; 56]
Site of measurement of glycemia*	**N (%) / Med [IQR]**
Capillary	638(8.3) / 95.7 % [85.7; 100]
Venous	61(9.4) / 0 % [0; 0]
Arterial	296(5.6) / 0 % [0; 11.1]
Subcutaneous continuous glucose sensor	15 (2.3) / 0 % [0; 0]
Number of glycemia measure per patient	14 [12; 19]
At least /4 h	550 (84.7)
At least /2 h	78 (12)
Care Burden	
Number of patients assigned per nurse (missing = 1)	2.3 [2; 2.8]
Workload score (missing = 4)	4.3 [3.2; 5.2]
Years of ICU experience for the nurse	4.8 [2.9; 7.4]

*At least once per patients and rates per patient.

BGC: blood control glucose; GLI: Glycemic Lability Index; GluCV: Glucose variability coefficient; ICU: intensive care unit.

**Table 3:** Comparison according to the blood glucose target group defined by protocol.

Glycemic targets	Tight BGC Upper range <7.8 mmol/L (1.4 g/L)	Standard BGC Upper range 7.8−10 mmol/L (1.4; 1.8 g/l)	Liberal BGC Upper range > 10 mmol/L (1.8 g/l)	*P value*
Number of patients	82	118	449	.
Age (year)	69 [59; 75]	69 [59; 76]	68 [60; 74]	0.72
Sex (Male)	52 (63.4)	71 (60.2)	304 (67.7)	0.27
BMI (kg/m²) (missing = 2)	26.1 [23; 29.7]	27.6 [24.5; 32]	27.7 [23.9; 32.7]	**0.03**
Type of admission				
Medical	57 (69.5)	102 (86.4)	388 (86.4)	**<0.01**
Urgent surgery	9 (11)	8 (6.8)	46 (10.2)	.
Scheduled surgery	16 (19.5)	8 (6.8)	15 (3.3)	.
SAPS II	42 [35; 55]	47.5 [33; 68]	48 [35; 59]	0.17
SOFA	7 [4; 9]	7 [4; 11]	6 [4; 9]	0.16
Feeding				
No	2 (2.4)	8 (6.8)	36 (8.0)	0.19
Glucose	71 (89.9)	75 (68.2)	335 (81.1)	**<0.01**
Enteral nutrition	43 (53.8)	61 (55.5)	255 (61.7)	0.26
Total parenteral nutrition	16 (20.3)	37 (33.6)	68 (16.5)	**<0.01**
Nutrition withdrawal	12 (14.6)	11 (9.3)	49 (10.9)	0.49
Acute liver failure	3 (3.7)	3 (2.5)	13 (2.9)	0.90
Sepsis	37 (45.1)	69 (58.5)	207 (46.1)	0.05
Steroids	27 (32.9)	49 (41.5)	204 (45.6)	0.09
SOFA H48	4 [3; 7]	5 [2; 9]	5 [2; 8]	0.55
ICU length of stay	8 [5; 12]	8 [5; 16]	8 [5; 14]	0.64
ICU death	14 (17.3)	31 (26.3)	76 (17)	0.07
Organ supports during ICU stay				
IMV	40 (49.4)	77 (65.3)	277 (61.8)	0.06
Vasopressors	49 (60.5)	76 (64.4)	278 (62.1)	0.84
RRT	10 (12.4)	22 (18.6)	65 (14.5)	0.41
Number of glycemia measures per patient	14 [11; 17]	14 [12; 17]	14 [13; 19]	0.07
At least /4 h	61 (74.4)	95 (80.5)	394 (87.8)	**<0.01**
At least /2 h	11 (13.4)	11 (9.3)	56 (12.5)	0.59
Max glycemia during ICU stay (mmol/L)	12.8 [9.7; 15.5]	13.9 [11.8; 17]	14.4 [12.2; 17.1]	**<0.01**
Min glycemia during ICU stay (mmol/L)	5.2 [4.4; 6.5]	6.3 [5; 7.2]	6 [4.8; 7.2]	**<0.01**
Adverse events				
At least one hypoglycemia <3.9 mmol/L	15 (18.3)	12 (10.2)	54 (12)	0.20
At least one severe hypoglycemia <2.2 mmol/L	6 (7.3)	4 (3.4)	8 (1.8)	**0.02**
At least one glycemia >11.1 mmol/L	51 (62.2)	99 (83.9)	390 (86.9)	**<0.01**
At least one glycemia >16.7 mmol/L	15 (18.3)	33 (28)	123 (27.4)	0.21
Glucose variability				
GLI ([mmol/L]^2^/hour/week)	1.5 [0.4; 2.8]	1.3 [0.7; 2.8]	1.5 [0.8; 2.9]	0.28
GluCV	22.8 [16.3; 34.7]	22.1 [16.2; 33.1]	25.2 [18.8; 33.1]	0.13
% time in the range	18.3 [8.7; 40]	27.5 [12.5; 44.2]	45.8 [27.1; 71.7]	**<0.01**
Insulin administration modalities				
At least once insulin IV	63 (76.8)	68 (57.6)	323 (71.9)	**<0.01**
At least once insulin bolus	37 (45.1)	73 (61.9)	225 (50.1)	**0.03**
At least once insulin long acting	6 (7.3)	5 (4.2)	31 (6.9)	0.55
Dose insulin /24 h	29.4 [11; 54.6]	31.1 [17; 61]	30 [13; 55.2]	0.59
On average per patient				
Number of patients assigned per nurse (missing = 1)	2.4 [2; 2.9]	2.5 [2.1; 3]	2.3 [2; 2.7]	**<0.01**
Workload score (missing = 4)	4.3 [2.8; 5.4]	4.6 [3.4; 5.3]	4.2 [3.1; 5.2]	0.13
Years of ICU experience for the nurse	3.8 [1.8; 8.5]	5 [2.8; 7.7]	4.9 [2.8; 7.6]	0.40
Number of glycemia measurements	1276 (100)	1785 (100)	7294 (100)	.
Deviation from protocol	318 (25)	456 (25.6)	1801 (24.7)	0.75

Results expressed as number (percentage) or median (interquartile range) unless otherwise specified.

BGC: blood glucose control; BMI: body mass index; GLI: glycemic lability index; GluCV: Glucose coefficient of variability; BMI: body mass index; SAPS II: Simplified acute physiology score; SOFA: Sequential Organ Failure Assessment; ICU: intensive care unit; IMV: invasive mechanical ventilation, RRT: renal replacement therapy; BG: blood glucose.

**Fig. 1:**
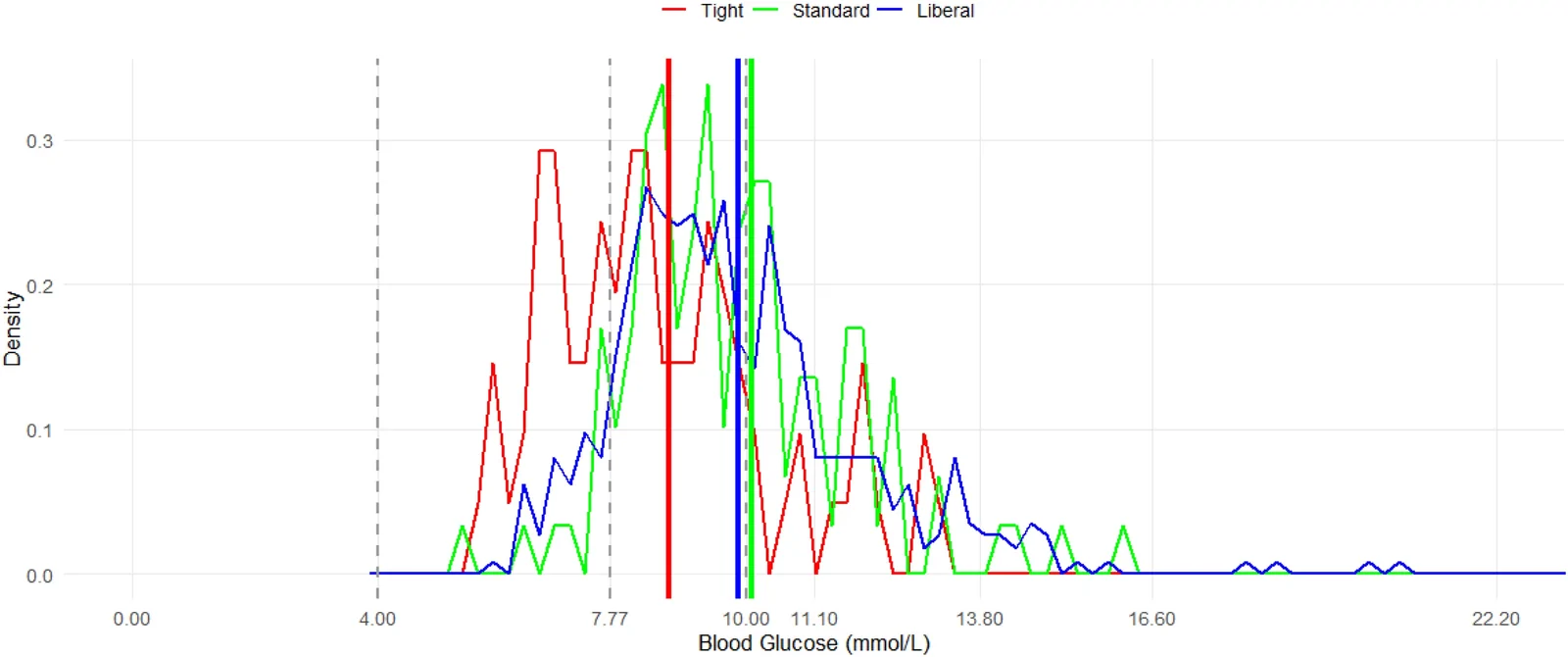
Distribution of blood glucose by target with solid vertical lines indicate mean blood glucose.

### Characteristics of glycemic variability during study protocol

Glycemic variability measures, characterized by GLI index and gluCV were respectively 1.5 [0.7; 2.9] (mmol/L)^2^/h/week and 24.6 [17.9; 33.2] (Table 2). Glycemic variability indices, including maximum glycemia, GLI, gluCV, and average glycemia, were strongly correlated (Figure S1). GLI values categorized by quartiles are presented in Table 4. Almost all patients in the highest GLI quartile had hyperglycemia (>11.1 mmol/L), hypoglycemia was rare in the lowest quartile but increased markedly across GLI quartiles, reaching 34.6% in the highest quartile (overall *p* <  0.01). Efficacy illustrated with the percentage of time spent in the range of prescribed glucose target significantly decreased with GV. In univariable logistic regression analyses, the occurrence of hypoglycemic <3.9 mmol/L (0.7 g/L) and hyperglycemic >11.1 mmol/L (2 g/L) episodes was strongly associated with increased GLI values (Table 5, OR 10.67, 95% CI [6.48–17.58] and OR 18.04, 95% CI [11.19–29.08], respectively; p < 0.01). Furthermore, GV significantly increased with the presence of diabetes and chronic renal failure with OR 2.72 (95% CI [2.05–3.62], *p* < 0.01) and 1.81 (95% CI [1.26–2.59], *p* < 0.01) respectively.

**Table 4:** Characteristics according glycemic variability expressed with GLI quartile.

Variables (Median [IQR]/N(%))	GLI Q1	GLI Q2	GLI Q3	GLI Q4	*P value*
Number of patients	163	161	163	162	.
Age	67 [58; 73]	67 [60; 74]	69 [59; 75]	69 [62; 74]	*0.21*
Sex (Male)	104 (63.8)	109 (67.7)	110 (67.5)	104 (64.2)	*0.82*
BMI (kg/m²) (miss = 2)	27.3 [24.1; 31.6]	28.2 [24.4; 32.7]	26.7 [23.9; 32]	27.4 [23.5; 31.2]	*0.54*
Type of admission					
Medical	119 (73.0)	141 (87.6)	138 (84.7)	149 (92.0)	***<0.01***
Urgent surgery	23 (14.1)	14 (8.7)	17 (10.4)	9 (5.6)	.
Scheduled surgery	21 (12.9)	6 (3.7)	8 (4.9)	4 (2.5)	.
Main reason for admission					
Respiratory	51 (31.3)	75 (46.6)	72 (44.2)	81 (50)	*0.06*
Neurological	24 (14.7)	23 (14.3)	24 (14.7)	10 (6.2)	.
Cardiovascular	33 (20.2)	17 (10.6)	21 (12.9)	26 (16.0)	.
Digestive / hepatic	20 (12.3)	16 (9.9)	14 (8.6)	7 (4.3)	.
Urological / nephrological	6 (3.7)	10 (6.2)	10 (6.1)	11 (6.8)	.
Metabolic / endocrinological	2 (1.2)	1 (0.6)	2 (1.2)	4 (2.5)	.
Hematological	4 (2.5)	4 (2.5)	3 (1.8)	1 (0.6)	.
Gynecological-obstetric	1 (0.6)	1 (0.6)	1 (0.6)		.
ENT	4 (2.4)		3 (1.8)	2 (1.2)	.
Other	18 (11.0)	14 (8.7)	13 (8.0)	20 (12.3)	.
Main comorbidities					
Cardiovascular	40 (24.5)	37 (23.0)	40 (24.5)	45 (27.8)	*0.79*
Chronic respiratory failure	35 (21.5)	41 (25.5)	37 (22.7)	45 (27.8)	*0.55*
Immunosuppression	24 (14.7)	28 (17.4)	24 (14.7)	31 (19.1)	*0.65*
Chronic liver failure	10 (6.1)	14 (8.7)	14 (8.6)	10 (6.2)	*0.69*
Chronic renal failure	18 (11.0)	27 (16.8)	32 (19.6)	40 (24.7)	***0.01***
Diabetes	47 (28.8)	64 (39.75)	92 (56.4)	103 (63.6)	***<0.01***
Severity at admission					
SAPS II	46 [34; 58]	50 [35; 64]	47 [35; 62]	44 [30; 57]	*0.12*
SOFA	7 [4; 10]	7 [4; 10]	7 [4; 10]	5 [3; 8]	***<0.01***
SOFA respiratory >2	62 (38.0)	57 (35.4)	65 (39.9)	48 (29.6)	*0.24*
SOFA cardio-vascular >2	83 (50.9)	73 (45.3)	75 (46.0)	55 (34.0)	***0.02***
SOFA neurological >2	56 (34.4)	59 (36.6)	60 (36.8)	29 (17.9)	***<0.01***
SOFA kidney >2	26 (15.95)	29 (18.01)	20 (12.27)	25 (15.43)	*0.55*
Feeding					
No	10 (6.1)	13 (8.1)	10 (6.1)	13 (8.0)	*0.83*
Glucose	136 (88.9)	109 (73.6)	114 (74.5)	122 (82.4)	***<0.01***
Enteral nutrition	86 (56.2)	91 (61.5)	89 (58.2)	93 (62.4)	*0.67*
Total parenteral nutrition	30 (19.6)	34 (23.0)	30 (19.6)	27 (18.2)	*0.77*
Nutrition withdrawal	11 (6.7)	20 (12.4)	25 (15.3)	16 (9.9)	*0.09*
Acute liver failure	5 (3.1)	7 (4.3)	4 (2.5)	3 (1.9)	*0.58*
Sepsis	72 (44.2)	89 (55.3)	84 (51.5)	68 (42.0)	*0.06*
Steroids	55 (33.7)	85 (52.8)	74 (45.7)	66 (41.0)	***<0.01***
ICU length of stay (miss = 2)	8 [6; 15]	8 [5; 14]	8 [5; 15]	7 [4; 11]	***0.03***
ICU death	30 (18.5)	31 (19.3)	34 (20.9)	26 (16.1)	*0.75*
Organ Support during ICU stay					
IMV	110 (67.9)	102 (63.4)	109 (66.9)	73 (45.3)	***<0.01***
Vasopressors	115 (71.0)	105 (65.2)	104 (63.8)	79 (49.1)	***<0.01***
RRT	18 (11.1)	29 (18.0)	22 (13.5)	28 (17.4)	*0.25*
Blood glucose management					
Number of glycemia measurements	13 [12; 15]	14 [12; 19]	15 [13; 19]	17 [13; 23]	***<0.01***
At least /4 h	125 (76.7)	133 (82.7)	143 (87.7)	149 (92.0)	***<0.01***
At least /2 h	3 (1.8)	16 (9.9)	23 (14.1)	36 (22.2)	***<0.01***
Median glycemia per patient (mmol/L)	8.3 [7.8; 9.4]	9.4 [8.3; .4]	10 [8.9; 11.1]	10.5 [8.9; 14]	***<0.01***
Max glycemia during ICU stay (mmol/L)	11.2 [9.9; 12.6]	13.3 [11.8; 14.8]	15.3 [13.5; 17.5]	18.1 [16.1; 22.2]	***<0.01***
Min glycemia during ICU stay (mmol/L)	6.5 [5.6; 7.2]	6.2 [5.4; 7.3]	5.9 [4.8; 7.3]	4.6 [3.4; 6]	***<0.01***
Adverse events					
At least one hypoglycemia (< 3.9 mmol/L)	1 (0.6)	5 (3.1)	19 (11.7)	56 (34.6)	***<0.01***
At least one severe hypoglycemia (< 2.2 mmol/L)	0	0	4 (2.5)	14 (8.6)	***<0.01***
At least one hyperglycemia (> 11.1 mmol/L)	82 (50.3)	137 (85.1)	160 (98.2)	161 (99.4)	***<0.01***
At least one severe glycemia (> 16.7 mmol/L)	1 (0.6)	14 (8.7)	51 (31.3)	105 (64.8)	***<0.01***
Tight glycemic control (upper range <7.8 mmol/L)	29 (17.8)	12 (7.5)	22 (13.5)	19 (11.73)	***0.045***
% time in the range	54.17 [26.67; 87.3]	42.86 [22.92; 71.7]	34.92 [19.77; 58.3]	30.35 [16.67; 43.8]	***<0.01***
Insulin therapy					
At least once insulin IV	83 (50.92)	117 (72.67)	122 (74.85)	132 (81.48)	***<0.01***
At least once insulin bolus	84 (51.53)	76 (47.2)	89 (54.6)	86 (53.09)	*0.58*
At least once insulin long acting	5 (3.07)	7 (4.35)	10 (6.13)	20 (12.35)	***<0.01***
Dose insulin /24 h	15 [4.5; 32.5]	25 [12.36; 51]	35 [22; 65.5]	45.25 [22; 75.58]	***<0.01***
Site of measurement of glycemia					
Capillary	158 (96.93)	159 (98.76)	162 (99.39)	159 (98.15)	*0.36*
Venous	10 (6.13)	8 (4.97)	19 (11.66)	24 (14.81)	***<0.01***
Arterial	73 (44.79)	82 (50.93)	72 (44.17)	69 (42.59)	*0.46*
Transcutaneous	1 (0.61)	3 (1.86)	3 (1.84)	8 (4.94)	*0.06*
Care burden					
Number of patients assigned per nurse (missing = 1)	4.31 [3.25; 5.09]	4.33 [3.24; 5]	4.34 [3.25; 5.38]	4.18 [3.08; 5.30]	*0.23*
Workload score (missing = 4)	4.62 [2.62; 7.07]	5.55 [3.25; 8]	4.79 [2.92; 7.33]	4.44 [2.69; 7.07]	*0.77*
Years of ICU experience for the nurse	2.35 [2; 2.692]	2.29 [2; 2.75]	2.38 [2.05; 2.92]	2.28 [2; 2.895]	*0.27*
Number of glycemia measurement	2492 (100)	2419 (100)	2560 (100)	2884 (100)	.
Deviation from protocol	570 (22.9)	549 (22.7)	685 (26.8)	771 (26.7)	***<0.01***

GLI: glycemic lability index; BMI: body mass index; SAPS II: Simplified acute physiology score; SOFA: Sequential Organ Failure Assessment; ICU: intensive care unit; ENT: ear, nose, throat, IMV: invasive mechanical ventilation, RRT: renal replacement therapy; BG: blood glucose.

**Table 5:** Factors associated with increased GLI value (univariate analyses).

Variables	OR [CI 95%]	*P value*
Age	1.01 [1–1.03]	*0.02*
Sex (Male)	1.01 [0.75–1.35]	*0.95*
BMI (kg/m²)	0.99 [0.97–1.01]	*0.43*
Main comorbidities		
Cardiovascular	1.13 [0.82–1.56]	*0.46*
Chronic respiratory failure	1.19 [0.86–1.64]	*0.28*
Immunosuppression	1.17 [0.8–1.7]	*0.41*
Chronic liver failure	1 [0.6–1.67]	*1.00*
Chronic renal failure	1.81 [1.26–2.59]	*<0.01*
Diabetes	2.72 [2.05–3.62]	*<0.01*
Characteristics on admission		
SAPS II	1 [0.99–1]	*0.19*
SOFA	0.94 [0.91−0.98]	*<0.01*
SOFA respiratory >2	0.84 [0.63–1.11]	*0.22*
SOFA cardio-vascular >2	0.66 [0.5−0.88]	*<0.01*
SOFA neurological >2	0.64 [0.48−0.86]	*<0.01*
SOFA kidney >2	0.89 [0.61–1.31]	*0.56*
Acute liver failure	0.69 [0.32–1.53]	*0.37*
Sepsis	0.92 [0.7–1.21]	*0.56*
Steroids	1.13 [0.86–1.49]	*0.39*
Feeding		
No	1.12 [0.66–1.92]	*0.68*
Glucose	0.8 [0.57–1.13]	*0.20*
Enteral nutrition	1.14 [0.85–1.52]	*0.39*
Total parenteral nutrition	0.91 [0.64–1.3]	*0.62*
Variables related to glycemia		
At least /4 h	2.22 [1.51–3.27]	<0.01
At least /2 h	3.44 [2.23–5.31]	<0.01
Tight blood glucose control (*vs* standard and liberal groups)	0.79 [0.51–1.21]	0.28
Adverse events		
At least one hypoglycemia (<3.9 mmol/L)	10.67 [6.48–17.58]	*<0.01*
At least one hyperglycemia (>11.1 mmol/L)	18.04 [11.19–29.08	*<0.01*
At least once insulin IV	2.56 [1.87–3.49]	*<0.01*
At least once insulin bolus	1.1 [0.84–1.45]	*0.49*
Variables related to nurses		
Number of patients assigned per nurse	1.25 [0.97–1.61]	*0.08*
Workload score	0.98 [0.89–1.08]	*0.63*
Years of ICU experience for the nurse	0.99 [0.96–1.03]	*0.78*

SAPS II: Simplified acute physiology score; SOFA: Sequential Organ Failure Assessment; ICU: intensive care unit; IV: intravenous.

### Primary outcome

Management with tighter glucose targets was not associated with increased GLI in univariable analysis (OR 0.79, 95% CI [0.51–1.21], *p* = 0.28) (Table 5, Fig. 2). Among the three groups, standard glucose target was associated with the lowest GV, although the difference was not significant. (Table 3).

**Fig. 2:**
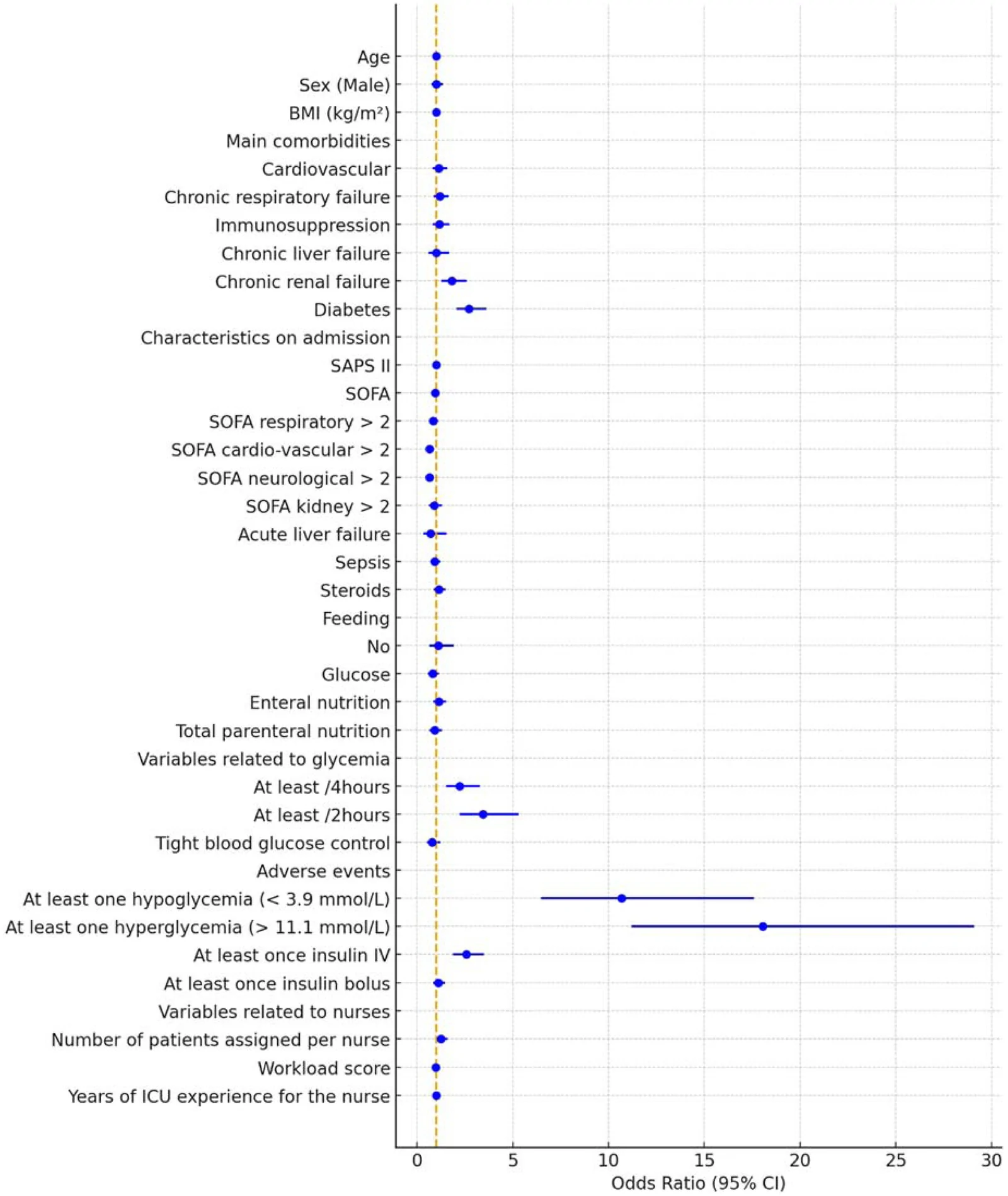
Factors associated with increased GLI value in univariate analyses.

### Secondary outcomes

Severe hypoglycemic events were more frequent in the tight glycemic target group than in the other two (*p* < 0.02) (Table 3). On the contrary, hyperglycemia >11.1 mmol/L occurred more often in the liberal group (*p* < 0.01). The percentage of time spent in the target range was significantly higher in the liberal group (45.8% vs. 18.3% in the tight control group, *p* < 0.01).

***Management protocol and care workload***. Insulin administration was more frequent in the tight target group (p < 0.01) (Table 3). More frequent glucose measurements (at least every 2 h, OR 3.44, 95% CI [2.23–5.31], *p* < 0.01) and increased insulin use, especially intravenous insulin (OR 2.56, 95% CI [1.87–3.49], p < 0.01), were also associated with higher GLI (Table 5, Fig. 2). Nurse workload did not significantly impact GV (*p* = 0.63). No significant difference in the perceived nurse workload was observed between the different target groups.

***Adherence to the protocol***. A total of 2575 (24.9%) deviations from the protocol were recorded. No difference was observed in the reported number of protocol deviations according to the target group used, but number of them significantly increased with GLI quartiles (22.9% in Q1 vs. 26.7% in Q4, *p* < 0.01).

***Factors associated with morbidity and ICU death***. Comparison between ICU deaths and survivors revealed significant differences (Table S4). ICU mortality rate was significantly higher in patients who had at least one episode of hypoglycemia below 3.9 mmol/L (0.7 g/L) (*p* = 0.04), whereas this rate was less frequent in patients with at least one episode of hyperglycemia >11.1 mmol/L (2 g/L) (*p* = 0.04). However, there were no significant differences in GV indices. ICU deaths were significantly linked to higher nurse workload scores wit reported mean score at 4.2 vs 4.7 in alive and dead patients respectively (*p* < 0.01).

## Discussion

This study provides insights on glycemic monitoring based on data from one of the largest multicenter ICU prospective cohorts to date. We highlighted that management with tight glucose control protocols was not associated with increased GV compared with more liberal target strategies. Management with standard glycemic target (blood glucose upper range between 7.8 and 10 mmol/L) seemed to be associated with lower GV without being statistically significant. We noted that higher GV (GLI) was significantly associated with diabetes and chronic renal failure. In contrast, cardiovascular and neurological failure at ICU admission were inversely associated with increased GLI. Although higher GLI was related to a greater incidence of dysglycemias, it was not associated with an increased ICU mortality. Nurse workload was not significantly associated with GV, and perceived workload did not differ according to blood glucose targets groups. We also observed substantial heterogeneity in practices both between and within ICUs.

These findings warrant several comments. First, the heterogeneity observed in glycemic control practices, as recently reported by Desgrouas and colleagues, encompassed multiple aspects [37]. Differences between ICUs primarily concerned blood glucose targets, but also extended to measurement sites and insulin therapy protocols. A 25% rate of protocol deviation, was reported by nurses raising questions about adherence to the local protocol and the factors contributing to these discrepancies. These non-consensual practices highlight the ongoing uncertainty in the literature regarding the optimal glucose target. American guidelines recommend that ICU physicians initiate glycemic management protocols and procedures to treat persistent hyperglycemia greater than or equal to 10 mmol/L (1.8 g/L) in critically ill adults [19]. This recommendation reflects the lack of studies evaluating the optimal blood glucose threshold for initiating insulin infusion therapy in critically ill adult patients, independently of the target glucose range. Experts recommend utilization of protocols that minimize the risk of hypoglycemia and emphasize timely treatment. Moreover, based on available randomized controlled trials, the scientific community recommends against insulin infusion targeting a lower blood glucose range (4.4–7.7 mmol/L) compared to a higher blood glucose range (7.8–11.1 mmol/L), to reduce the risk of hypoglycemia [19]. In the context of French recommendations, experts suggest to avoid hyperglycemia upper than 10 mmol/L [17]. For neurotrauma patients, a glucose goal that avoids hypoglycemia while targeting levels <10 mmol/L (1.8 g/L) should be acceptable. More recently, Gunst et al. proposed a practical framework for glucose control in the ICU [31]. According to these experts, optimal blood glucose target should vary depending on the clinical situation. If a validated protocol is available, with demonstrated high time in glucose target range and a minimal risk of hypoglycemia, tight glucose control between 4.4–6.1 mmol/L (0.8–1.1 g/L) can be considered. Otherwise, a more moderate strategy aiming to maintain blood glucose value below 8.3−10 mmol/L (1.5–1.8 g/L) is recommended. Second, our study found that tight glucose control protocols, compared to standard and liberal protocols, were not associated with lower GV. To our knowledge, there is no study reporting a relationship between glycemic targets and GV. The main hypothesis for this result could have been that we observed no significant difference between target groups concerning the number of glucose measurements per patient during study period. However, we could note that performing frequent blood glucose measurements was associated with increased GV (every 2 and 4 h). Furthermore, as previously reported, external confounding factors—such as corticosteroid therapy—may have contributed to increased GV in our cohort by exacerbating hyperglycemia. Third, and according to the literature, we noted a significant increase in the risk of severe hypoglycemia in the tight glucose control group with upper range < 7.8 mmol/L (1.4 g/L). Lastly and surprisingly, patient severity, illustrated by higher cardiovascular and neurological SOFA at admission, was found to be a factor associated with a decrease of GV. However, a recent one-day study has reported that higher glycemia was associated with a higher SOFA score at the study day [37]. These results raise several important points. In patients with high cardiovascular and neurological SOFA scores, early enteral nutrition may be delayed or contraindicated. This may have interfered with GV. Nutritional strategies in critically ill patients have shown significant variability in outcomes, as highlighted by landmark studies such as EPANIC [38], NUTRIREA-III [39], and the secondary analysis by Fivez et al. [40]. These trials underscore the complexity of early versus delayed parenteral nutrition and its impact on clinical outcomes, including glycemic control. For instance, in the NUTRIREA III study, lower caloric intake (approximately 6 kcal/kg/day) was associated with improved glycemic stability. Blood glucose levels were more consistently maintained between 8 and 10 mmol/L in the low-calorie group, with a daily insulin requirement of 30–40 IU, compared to 10–12 mmol/L and 45–55 IU of insulin in the higher-calorie intervention group. These findings suggest that reduced nutritional intake may facilitate better glycemic control, potentially contributing to improved metabolic stability in the early phase of critical illness [41]. Another hypothesis is that greater patient severity at the acute phase may prompt more intensive monitoring, leading to increased vigilance in glycemic management. Interestingly, increased GV was found to be associated with several comorbidities. Unlike sepsis, diabetes and chronic renal failure were specifically linked to higher GV. This observation confirms previous results showing an association between GV (with several indices) and the presence of diabetes mellitus [36]. Similarly, subgroup analysis of other glycemic parameters revealed a significant increase in the number of hyperglycemic and hypoglycemic episodes in this diabetic population (Table S5). Although the CONTROLING study found no benefit of hemoglobin A1c-guided glucose control, partly due to significant overlap glucose levels between groups [20], international guidelines suggest a potential benefit of personalized glucose targets, aligned with chronic levels on glycemia. As Donati and colleagues, we also observed an increase of mean glucose value per patient was associated with high GV [36]. Finally, no association between GV and nursing workload was found, despite the fact that increase of glycemic variability was linked to more frequent glucose measurements. Notably, we noted that the choice of blood glucose control strategy had no impact on the nurses’ perception of their workload.

## Limitations

Several limitations of this study should be acknowledged. First, this study was multicentric and international but reflects only French-speaking practices for glucose management. Second, because of the study design, which limited glucose management data collection to a 48 -h period, the number of glucose measurements was reduced, potentially affecting the assessment of GV. However, compared to other practice surveys, our study collected a total of 10355 glycemia measurements in 649 patients over the two-day period, making the results relevant and representative of ICU patients. More precisely regarding the observed practices, although insulin bolus administration was frequently reported, the route of administration and the specific indication for these boluses were not collected, limiting our ability to characterize this practice further. We explored the first 48 h of glucose management, a period typically marked by a hypercatabolic state and stress-induced hyperglycemia corresponding to the acute phase of critical illness. Persistent high GV after this acute phase may reflect insulin resistance, potentially related to undiagnosed diabetes or ongoing and uncontrolled acute illness. Third, due to the disparities in prescribed glycemic targets, we attempted to establish three blood glucose target profiles according to the literature and expert opinions. Nevertheless, the heterogeneity and significant imbalance among number of patients between groups calls for cautious interpretation of our results. Fourth, regarding the study population, the prevalence of diabetes in our cohort was high (47.1%) compared with that generally reported in unselected critically ill populations. As the study specifically enrolled patients with hyperglycemia requiring insulin therapy within the first 48 h after ICU admission, patients with pre-existing diabetes were therefore more likely to meet the eligibility criteria and to be included. Consequently, our cohort represents ICU patients requiring early glucose control rather than an unselected critically ill population, which may limit the generalizability of our findings. Similarly, in our cohort, both diabetes mellitus (OR 2.72, 95% CI [2.05–3.62]) and intravenous insulin administration (OR 2.56, 95% CI [1.87–3.49]) were associated with higher GV in univariable analyses. However, these associations should be interpreted cautiously, as patients with diabetes may have greater insulin requirements, while patients with greater glucose dysregulation are also more likely to require intensive insulin therapy. The association between insulin therapy and GV we observed cannot be interpreted as a direct effect of insulin therapy, and the interrelationship between diabetes status, insulin requirements, and GV may influence the observed associations. Moreover, although the last guidelines for glucose management included this point, glycosylated hemoglobin A1c was not included in the analyses because of substantial missing data. Finally, contrary to the literature, we found no association between ICU mortality and GV [42]. This may primarily be explained by the short duration of our study. In the cohort of Donati et al., GV was calculated during all the ICU length of stay with a mean duration of 4 days [36]. However, as expected, we found an association between ICU mortality and the occurrence of at least one episode of hypoglycemia. This result, in accordance with others, strengthens guidelines statements to pay strong attention to avoid the occurrence of hypoglycemia in ICU patients.

## Perspectives

Identifying the best glucose target range in order to reduce heterogeneity practices in ICUs remains the main objective for current practice. As suggested by international guidelines, a better personalized glucose target that considers prehospital glycemic status may be a key point for the future. As illustrated in our study, higher GV was associated with diabetes and renal failure at admission. In this way, future studies could focus on the impact of individualized glucose control on GV, and ultimately on ICU mortality. Also, to prevent severe hypoglycemia, developing well-performing algorithms for managing pronounced hyperglycemia—especially when aggravated by iatrogenic factors—appears to be crucial.

## Conclusion

Due to a lack of strong recommendations on glycemic targets, heterogeneity in prescription of glycemic objectives persists, even when protocols are available in ICUs. Glycemic management with tighter targets does not reduce GV, but results in more frequent severe hypoglycemic events which are associated with ICU mortality.

## Authors’ contributions

SG: conception and design. Data acquisition and interpretation. Manuscript draft. TK: conception and data manager. CD and BH: Manuscript draft and statistical analysis. LP: conception and design. Others: data acquisition, critical revision of the manuscript for intellectual content.

## Consent of publication

Not applicable.

## Ethics approvals and consent to participate

This study was approved by the Comité de Protection des Personnes Sud Est V in January 2023.

## Funding

Not applicable

## Availability of data and materials

The data sets of this study are available from the corresponding authors on reasonable request.

## Declaration of competing interest

Not applicable.
