# Supplementary material for: Beyond blood glucose targets: integrating glucose variability into acute glycemic care in French ICUs - a multicenter prospective study
Source: Ann Intensive Care. 2026 Oct 28;16:100193. doi: 10.1016/j.aicoj.2026.100193 (PMC13756784; doi:10.1016/j.aicoj.2026.100193)
Supplement: Supplementary file 1 [file mmc1.docx]

Table S 1: Characteristics of glucose management in 36 French-speaking ICUs

| Characteristics | N (%) |
| --- | --- |
| Existence of a department protocol | 35 (97.2) |
| Glycemic targets defined in protocols | 31 (86.1) |
| Blood glucose target  Tight: upper range <7.8 mmol/L (1.4 g/L)  Standard: upper range 7.8-10 mmol/L (1.4-1.8 g/l)  Liberal: upper range > 10 mmol/L (1.8 g/l) | 4/31 (13)  9/31 (29)  18/31 (58.1) |
| Existence of a blood glucose meter per patient | 32 (88.6) |
| Preferred blood glucose measurement site  Capillary in first intention  Arterial in second intention | 32 (88.9)  17 (47.2) |
| Blood sugar measurements taken by nursing assistants | 26 (72.2) |
| “Dynamic protocol” with variable glycemic measurement frequencies | 15 (41.7) |
| Protocol with dedicated central catheter line for insulin infusion | 11 (30.6) |
| Preferred line for insulin in the protocol  Median  Distal  Indifferent | 14 (38.9)  13 (36.1)  9 (25) |
| Maximum duration of insulin syringe predefined by the protocol  12 hours  24 hours  48 hours | 31 (86.1)  3 (8.3)  25 (69.4)  3 (8.3) |

Table S 2: Characteristics according to measures of glycemia

| Characteristics | Median [IQR]/N (%) |
| --- | --- |
| Number of glycemia measurements | 10355 |
| Mean glycemia (mmol/L) | 8.9 [7.2; 11.7] |
| Number of hypoglycemia (< 3.9 mmol/L) | 143 (1.4) |
| Number of severe hypoglycemia (< 2.2 mmol/L) | 24 (0.2) |
| Number of hyperglycemias (> 11.1 mmol/L) | 2762 (26.7) |
| Number of severe glycemia (> 16.7 mmol/L) | 474 (4.6) |
| Site of measurement of glycemia |  |
| - Capillary | 8998/10348 (87) |
| - Venous | 154/10348 (1.5) |
| - Arterial | 1040/10348 (10.1) |
| - Subcutaneous continuous glucose sensor | 156/10348 (1.5) |
| Insulin administration modality |  |
| - Continuous IV / Injection rate (UI/h) | 5932 (57.3) / 2 [1 ; 4] |
| - Bolus / dose (UI) | 1683 (16.3) / 5 [3 ; 8] |
| Route of administration of the bolus |  |
| - Intravenous | 238/1683 (13.9) |
| - Subcutaneous | 1470/ 1683 (86.1) |
| Long-acting insulin / dose (UI) | 73 (0.7) / 16 [10 ; 24] |
| Deviation from protocol | 2575 (24.9) |

Table S 3: Characteristics during intervention and at the end of ICU stay

| *During 48 hours study period* | Median [IQR]/N(%) |
| --- | --- |
| Feeding |  |
| - No | 46 (7.1) |
| - Glucose | 481/602 (79.9) |
| - Enteral nutrition | 359/603 (59.5) |
| - Total parenteral nutrition | 121/602 (20.1) |
| Nutrition withdrawal | 72 (11.1) |
| Cumulated time for nutrition withdrawal (hours) (miss=585) | 22.5 [8.5; 28.5] |
| Motif for nutrition withdrawal |  |
| - Transport and/or surgery | 15/72 (20.8) |
| - Intubation and/or extubation | 27/72 (37.5) |
| - Abdominal surgery | 10/72 (13.9) |
| - Digestive intolerance | 16/72 (22.2) |
| - Others | 22/72 (30.6) |
| Acute liver failure | 19 (2.9) |
| Sepsis | 313 (48.2) |
| Steroids | 280 (43.3) |
| *At the end of the 48 hours period* |  |
| SOFA | 5 [2; 8] |
| - SOFA respiratory > 2 | 151 (23.3) |
| - SOFA neurological > 2 | 164 (25.3) |
| - SOFA cardio-vascular > 2 | 181 (27.9) |
| - SOFA liver > 2 | 12 (1.8) |
| - SOFA kidney > 2 | 84 (12.9) |
| - SOFA coagulation > 2 | 32 (4.9) |
| ICU Length of stay (miss=2) | 8 [5; 14] |
| ICU death | 121/647 (18.7) |
| Organ supports |  |
| Invasive mechanical ventilation during ICU stays | 394/647 (60.9) |
| Vasopressors during ICU stay | 403/647 (62.3) |
| ECLS during ICU stay | 11/647 (1.7) |
| RRT during ICU stay | 97/647 (15) |

SOFA: Sequential Organ Failure Assessment; ICU: intensive care unit; ECLS: extra corporeal live support; RRT: renal replacement therapy

Figure S 1: correlation matrix including patient characteristics.

GluCV: Glucose coefficient of variability; GLI: glycemic lability index; avg: average; ICU LOS: intensive care unit length of stay; SOFA: Sequential Organ Failure Assessment; SAPS 2: Simplified acute physiology score 2; BMI: body mass index.

Table S 4: Factors associated with vital status at ICU discharge

|  | Alive | Dead | *P value* |
| --- | --- | --- | --- |
| Age | 68 [59 ; 74] | 70 [62 ; 76] | *<0.01* |
| Sex (Male) | 337 (64.1) | 89 (73.6) | *0.05* |
| BMI (kg/m²) (miss=2) | 27.5 [23.9 ; 32] | 26.6 [23.2 ; 31.3] | *0.17* |
| Type of admission |  |  |  |
| -             Medical | 438 (83.3) | 107 (88.4) | *0.29* |
| -             Urgent surgery | 53 (10.1) | 10 (8.3) | *.* |
| -             Scheduled surgery | 35 (6.7) | 4 (3.3) | *.* |
| Main comorbidities |  |  |  |
| -             Cardiovascular | 128 (24.3) | 34 (28.1) | *0.39* |
| -             Chronic respiratory failure | 129 (24.5) | 27 (22.3) | *0.61* |
| -             Immunosuppression | 89 (16.9) | 17 (14) | *0.44* |
| -             Chronic liver failure | 39 (7.4) | 9 (7.4) | *0.99* |
| -             Chronic renal failure | 94 (17.9) | 22 (18.2) | *0.94* |
| -             Diabetes | 249 (47.3) | 57 (47.1) | *0.96* |
| Severity on admission |  |  |  |
| SAPS II | 44.5 [33 ; 57] | 56 [44 ; 71] | ***<0.01*** |
| SOFA | 6 [4 ; 9] | 8 [6 ; 12] | ***<0.01*** |
| Events and severity during ICU stay |  |  |  |
| Acute liver failure | 11 (2.1) | 8 (6.6) | ***<0.01*** |
| Sepsis | 242 (46) | 69 (57) | ***0.03*** |
| Steroids | 226 (43.1) | 52 (43) | *0.98* |
| SOFA H48 | 4 [2 ; 7] | 8 [6 ; 12] | ***<0.01*** |
| IMV | 287 (54.6) | 107 (88.4) | ***<0.01*** |
| Vasopressors | 298 (56.7) | 105 (86.8) | ***<0.01*** |
| RRT | 61 (11.6) | 36 (29.8) | ***<0.01*** |
| ICU length of stay (miss=2) | 8 [5 ; 13] | 9 [5 ; 21] | ***0.01*** |
| Blood glucose characteristics during study period |  |  |  |
| Median glycemia per patient (mmol/L) | 10.5 [8.3 ; 13.9] | 10 [7.8 ; 13.3] | *0.06* |
| Max glycemia during ICU stay (mmol/L) | 13.9 [12.2 ; 17.2] | 14.4 [11.7 ; 16.7] | *0.29* |
| Min glycemia during ICU stay (mmol/L) | 6.1 [5; 7.2] | 5.6 [4.4 ; 7.2] | *0.15* |
| At least one hypoglycemia (< 3.9 mmol/L) | 59 (11.2) | 22 (18.2) | *0.04* |
| At least one severe hypoglycemia (< 2.2 mmol/L) | 14 (2.7) | 4 (3.3) | *0.70* |
| At least one hyperglycemia (> 11.1 mmol/L) | 445 (84.6) | 93 (76.9) | ***0.04*** |
| At least one severe glycemia (> 16.7 mmol/L) | 141 (26.8) | 30 (24.8) | *0.65* |
| GLI ([mmol/L]^2^/hour/week) | 1.5 [0.7 ; 2.9] | 1.5 [0.7 ; 2.6] | *0.38* |
| gluCV | 24.4 [18 ; 32.9] | 25.7 [17.7 ; 36.6] | *0.30* |
| % time in the range | 39 [20 ; 64.4] | 42 [20.8 ; 71] | *0.19* |
| Dose insulin /24h | 29.2 [12.4 ; 54.5] | 33.5 [14 ; 59.5] | *0.17* |
| Nurse characteristics |  |  |  |
| Number of patients assigned per nurse (missing=1) | 2.4 [2 ; 2.8] | 2.3 [2 ; 2.9] | *0.33* |
| Workload score (missing=4) | 4.2 [3 ; 5.1] | 4.7 [3.8 ; 5.7] | ***<0.01*** |
| Years of ICU experience for the nurse | 4.7 [2.8 ; 7.6] | 5 [2.4 ; 7.3] | *0.38* |

BMI: body mass index; SOFA: Sequential Organ Failure Assessment; SAPS II simplified acute physiology score II; ICU: intensive care unit; IMV: invasive mechanical ventilation; RRT: renal replacement therapy; GLI: glycemic lability index; GluCV: glucose coefficient of variability.

*Table S5. Factors of glycemic variability associated with known diabetic status upon admission to the intensive care unit.*

| Variables | No diabetes | Diabetes | P value |
| --- | --- | --- | --- |
| Number of patients | 343 (100) | 306 (100) | . |
| Max glycemia during ICU stay (mmol/L) | **13.3 [11.1 ; 16.1]** | **15.5 [12.8 ; 18.3]** | **<.01** |

| Min glycemia during ICU stay (mmol/L) | 6.1 [5 ; 7.2] | 5.6 [4.4 ; 7.2] | 0.11 |
| --- | --- | --- | --- |

| At least one hypoglycemia (< 3.9 mmol/L) | 32 (9.3) | 49 (16) | 0.01 |
| --- | --- | --- | --- |
| At least one severe hypoglycemia (< 2.2 mmol/L) | **9 (2.6)** | **8 (2.6)** | **0.99** |
| At least one hyperglycemia (> 11.1 mmol/L) | **263 (76.7)** | **277 (90.5)** | **<.01** |
| At least one severe hyperglycemia (> 16.7 mmol/L) | **62 (18.1)** | **109 (35.6)** | **<.01** |
| GLI | **1.1 [0.6 ; 2.2]** | **2.1 [1.1 ; 3.9]** | **<.01** |
| GluCV | **22.1 [16.6 ; 30.3]** | **27.5 [20.3 ; 36.8]** | **<.01** |

ICU: intensive care unit; GLI: glycemic lability index; GluCV: glucose coefficient of variability
